# Comparative Chloroplast Genome Analysis of Rhubarb Botanical Origins and the Development of Specific Identification Markers

**DOI:** 10.3390/molecules23112811

**Published:** 2018-10-30

**Authors:** Yuxin Zhou, Jing Nie, Ling Xiao, Zhigang Hu, Bo Wang

**Affiliations:** 1 College of Pharmacy, Hubei University of Chinese Medicine, Wuhan 430065, China; zyx123hn@163.com; 2 Hubei Institute for Drug Control, Wuhan 430012, China; niejingwh@sina.com (J.N.); lingyun724@126.com (L.X.)

**Keywords:** rhubarb, *Rheum*, complete chloroplast genome, ultra-barcode, identification

## Abstract

Rhubarb is an important ingredient in traditional Chinese medicine known as *Rhei radix et rhizome*. However, this common name refers to three different botanical species with different pharmacological effects. To facilitate the genetic identification of these three species for their more precise application in Chinese medicine we here want to provide chloroplast sequences with specific identification sites that are easy to amplify. We therefore sequenced the complete chloroplast genomes of all three species and then screened those for suitable sequences describing the three species. The length of the three chloroplast genomes ranged from 161,053 bp to 161,541 bp, with a total of 131 encoded genes including 31 tRNA, eight rRNA and 92 protein-coding sequences. The simple repeat sequence analysis indicated the differences existed in these species, phylogenetic analyses showed the chloroplast genome can be used as an ultra-barcode to distinguish the three botanical species of rhubarb, the variation of the non-coding regions is higher than that of the protein coding regions, and the variations in single-copy region are higher than that in inverted repeat. Twenty-one specific primer pairs were designed and eight specific identification sites were experimentally confirmed that can be used as special DNA barcodes for the identification of the three species based on the highly variable regions. This study provides a molecular basis for precise medicinal plant selection, and supplies the groundwork for the next investigation of the closely related Rheum species comparing and correctly identification on these important medicinal species.

## 1. Introduction

*Rheum* (Polygonaceae), a genus containing eight sections and ~60 herbaceous species, is widely distributed in Asia, especially in the temperate and subtropical high mountainous regions [1]. Rhubarb (*Rhei radix et rhizome*), an important multi-origin traditional Chinese medicine, was first recorded in the Shennong Herbal Classic as *Jun Yao* due to its efficacy as an analgesic and anti-inflammatory, effective at clearing heat, removing toxicity, and improving blood stasis [2]. Modern pharmacology research suggests that rhubarb also exhibits anticancer, antiviral, hypotensive, and immune system regulatory effects [3]. At present, *R. palmatum*, *R. tanguticum*, and *R. officinale* are considered the legal species to be used to produce *Rhei radix et rhizome*, as recorded in the Chinese Pharmacopoeia 2015 edition.

In past reports, researchers have mainly focused on the extraction of bioactive components, chemistry, or pharmacology properties. However, as a typical multi-origin medicine, the composition of each pharmacological product is different with regard to the three *Rheum* species and its effect thus variable [4]. The source of the species plays a decisive role in the chemical composition of rhubarb [5]. The difference between the three rhubarb plants mainly lies in the degree of leaf division, whereby *R. officinale* leaves are lobed and broad triangular, the *R. palmatum* leaves are lobed and triangular, and *R. tanguticum* leaves are parted and lanceolate [6], The methods that have long served to identify rhubarb medicinal materials mainly adopt trait identification, microscopic identification, physical and chemical identification, but these methods depend on experience, their subjectivity is strong, and it is hard to distinguish between processed products and powders [7]. It is particularly important to accurately identify the species of rhubarb. Some scholars have used the *trnL*-*trnF* sequences of 13 species of the genus *Rheum* to analyze and design specific primer pairs for the identification of different botanical species of rhubarb [8]. *psbA-trnH* has also been used to distinguish rhubarb from the other 19 related Polygonaceae species [9]. The gene sequences of *matK* showed potential for the distinction of different *Rheum* sections [10]. From the results of the identification efficiency analysis, the identification success rate of *trnH-trnF* for *Rheum* is 84%, and the success rate for *matK* identification is 83.7%, indicating that the identification efficiency of the chloroplast genes is significantly higher than that of nuclear sequences, such as ITS2, for *Rheum*. Therefore, the current method can identifyf closely related *Rheum* species only to a certain degree.

Chloroplasts are ubiquitous in plant cells and play important roles in plants to carry out photosynthesis and energy conversion. The chloroplast genome is independent of nuclear genes and is dominated by maternal inheritance. The chloroplast genome of most angiosperms consists of four parts: a pair of inverted repeats (IRA and IRB), a large single-copy region (LSC), and one small single-copy region (SSC) [11]. With the expansion and contraction of the IR region, the chloroplast genome size is approximately 120~160 kb [12]. Numerous scholars suggest that the whole chloroplast genome sequence is an ideal genomic barcode because the genome size is moderate, the intraspecific sequences are relatively conservative, the interspecies variation is large, and the substitution rate is lower than nuclear genes but higher than mitochondrial genes [13].

In the present study, the chloroplast DNA of three different botanical species of rhubarb, the famous Chinese medicinal herb, were utilized. After assembly and annotation, the characteristics of the chloroplast genome were analyzed and the identification of a few specific short sequences which are easy to amplify and which contain identification sites to distinguish the study species. The current study laid the foundation of super barcode utilization in rhubarb, provided a molecular basis for precise medicinal plant selection, and supplied the groundwork for the next investigation of the closely related *Rheum* species comparing and correctly identification on these important medicinal species.

## 2. Results

### 2.1. Chloroplast Genome Features

The chloroplast genomes ranged from 161,053 bp (*R. tanguticum*) to 161,541 bp (*R. palmatum*) in length, and the chloroplast genomes of the three species shared the same GC content, 37.3%, which is similar to the reported chloroplast genome of angiosperms [14]. The GC content of the IR region is higher than that of LSC and SSC. These genome sequences have been submitted to GenBank with accession number MH572012 for *R. officinale*, and MH572013 for *R. tanguticum*. The sequence of *R. palmatum* is similar to the published sequence with accession number KR816224 [15] (Table 1).

The chloroplast genome of *Rheum* was found to encode 131 predicted functional genes, including 92 protein-coding genes, 31 tRNA genes, and eight rRNA genes (Table 2). Among them, seven genes, *trnL-TAG*, *trnI-GAT*, *rpl2*, *atpF*, *rpoC1*, *ndhA*, and *ndhB*, contain one intron; two genes contain two introns (*ycf3* and *clpP*); 18 genes have two copies; and one gene has three copies. Variation was observed among the different species; for example, *trnL-TAG* contains one intron in *R. officinale*, but none in *R. palmatum* and *R. tanguticum*. 

**Figure 1:**
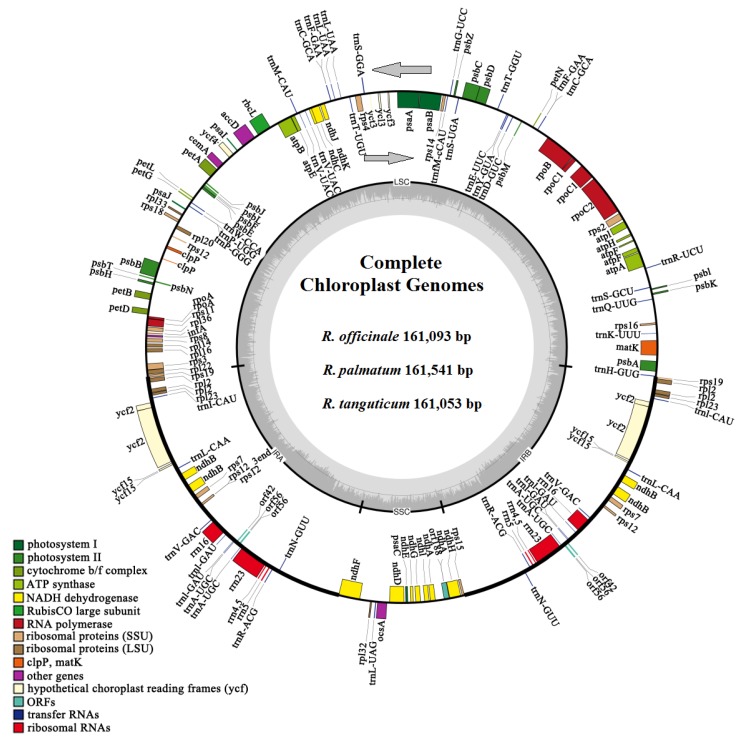
Gene map of the *Rheum* chloroplast genome. The genes lying inside and outside the outer circle are transcribed in a clockwise and counterclockwise direction, respectively (as indicated by arrows). Colors denote the genes belonging to different functional groups. The hatch marks on the inner circle indicate the extent of the inverted repeats (IRa and IRb) that separate the small single copy (SSC) region from the large single copy (LSC) region. The dark gray and light gray shading within the inner circle correspond to the percentage of G + C and A + T content, respectively.

Among the three rhubarb species, the gene rps19 is distributed in the LSC and IRA regions, and the gene ndhF is distributed in the IRA and SSC regions. In the *R. officinale* chloroplast genome, 62 protein-coding genes and 20 tRNA genes are located in the LSC region, and 10 protein-coding genes and one tRNA gene are located in the SSC region. In the chloroplast genome of *R. palmatum*, 62 protein-coding genes and 18 tRNA genes are located in the LSC region, and 10 protein-coding genes and two tRNA genes are located in the SSC region. In the chloroplast genome of *R. tanguticum*, 62 protein-coding genes and 18 tRNA genes are located in the LSC region, and 10 protein-coding genes and one tRNA gene are located in the SSC region. The chloroplast genes of the three species have a typical quadripartite structure (Figure 1).

### 2.2. Characterization of Simple Sequence Repeats

**Figure 2:**
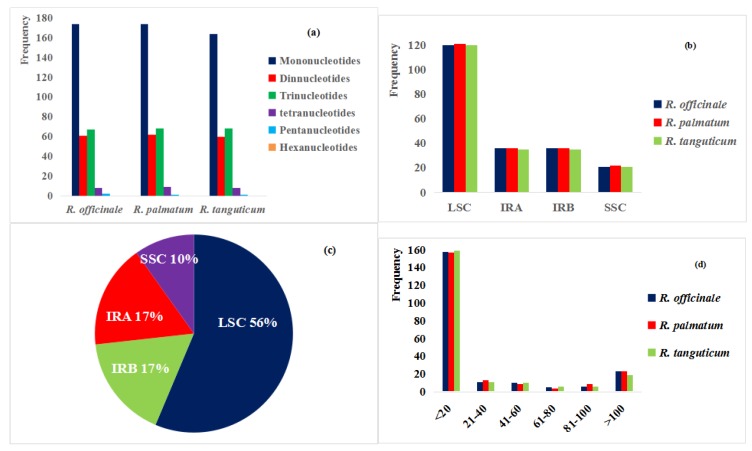
Comparison of SSR types and quantities in the three studied *Rheum* species. (**a**) Number of SSR types; (**b**) SSRs of three species in four regions; (**c**) The percentages of SSRs number in four regions; (**d**) Frequency of SSRs by length. SSR: Simple sequence repeats; LSC: large single-copy region; SSC: small single-copy region; IRA and IRB: inverted repeats.

The distribution of repeated sequences and the presence of the SSRs in the chloroplast genomes of the three species were analyzed. Among the three species, SSRs of *R. palmatum* (314) and *R. officinale* (312) were larger than SSR of *R. tanguticum* (301). There were 512, 183, 203, 25, and four mono-, di-, tri-, tetra- and pentanucleotide SSRs, respectively. No hexanucleotides were found in the three species. Among all SSRs, mononucleotide repeats were the most common, accounting for 55.23% of the SSR population, of which 507 A/T accounted for 99.0% of mononucleotides. The number of mononucleotides (174) is the same in *R. officinale* and *R. palmatum*; *R. palmatum* and *R. tanguticum* contain the same number of trinucleotides (68) and Pentanucleotides (1); there are 8 tetranucleotides in *R. officinale* and *R. tanguticum;* the number of dinucleotides in *R. officinale*, *R. palmatum* and *R. tanguticum* were 61, 62, and 60, respectively (Figure 2a).The SSRs of the three Rheum plants are similar in number in the four regions (Figure 2b), but the percentage of SSR in the four regions is different (Figure 2c). These findings show that SSRs are not evenly distributed in the chloroplast genomes. The length of most SSRs were <20 bp (Figure 2d). The distribution of *p*-type SSRs with a length greater than 10 bp was analyzed by using *R. officinale* as the representative (Table 3). The repeated sequences were mostly distributed in the non-coding sequences (CNS): intergenic spacers and intron regions, but found in coding regions (CDS), such as *rpoC2*, *petA*, *ycf2*, *ndhF*, *ndhG*, *matK*, *atpA*, *ndhD*, and *ycf1*. There is a 10 bp SSR between *petL* and the CNS. The others were similar to that of *R. officinale*, and are displayed in Supplementary Tables S1 and S2.

### 2.3. Phylogenetic Analysis

The phylogenetic relationships tree represents the results of a systematic study that can be used to describe the evolutionary relationships between species [16]. As can be seen from the neighbor joining tree, monocotyledons and dicotyledons were clustered together, and the support rate was 100%. Neighbor joining strongly supported the position of *Fagopyrum esculentum* and *F. tataricum* as a sister of the closely related species in the Polygonaceae. 

**Figure 3:**
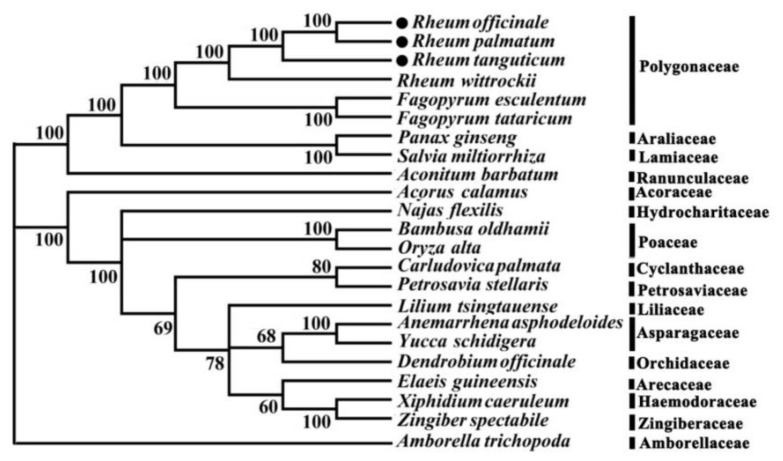
Phylogenetic tree constructed using neighbor joining (NJ), based on the whole chloroplast genomes from different species. *Amborella trichopoda* was set as outgroup.

The three species of rhubarb were clustered into a polytomy and *Rheum wittrockii* clustered together, indicating that despite the close relationship, the three species of rhubarb can be separated from each other via these regions (Figure 3). The chloroplast genome sequence provides a new method for the identification of *Rheum*. 

### 2.4. Comparative Genomic and Candidate Identification Sequence Analysis

**Figure 4:**
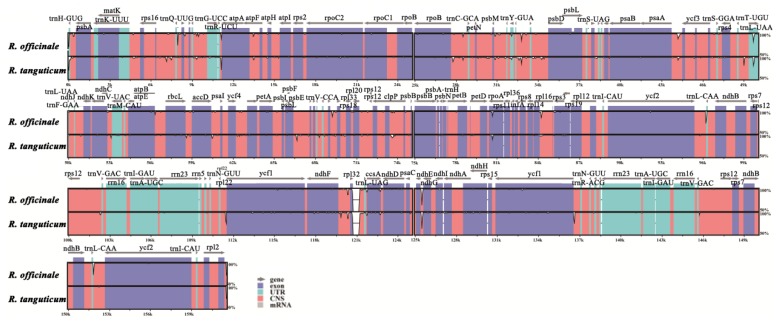
Comparison of three chloroplast genomes using *R. palmatum* as the reference. The vertical scale indicates the percentage of identity, ranging from 50% to 100%; the horizontal axis indicates the coordinates within the chloroplast genome. Annotated genes are displayed along the top. Genome regions are color-coded as either protein-coding exons, rRNA, tRNA, or conserved non-coding sequences (CNS). UTR: Untranslated Region.

The mVISTA [17] software was used to compare and analyze the chloroplast genomes of the three studied species in *Rheum*, and *R. palmatum* was used as a reference sequence. Overall, the IR region is more conservative than LSC and SSC, and coding regions were more conserved than non-coding ones. The genetic differences of the three species are mainly concentrated in the intron and intergenic spacers, and are mostly presented in the form of base substitution. For example, the gene *ycf3* and *clpP* both have two introns, while two sites are substituted in the *ycf3* exon, and six sites are replaced in *clpP*. Intergenic spacers differences existed in the genes, such as *psaA-ycf3*, *trnD-trnT*, *psbD-trnT*, *rpl16-rps3*, and *ccsA-ndhD*. The gene *accD* and *rpoC2* with two and eight base substitution, respectively, have no intron (Figure 4).

**Figure 5:**
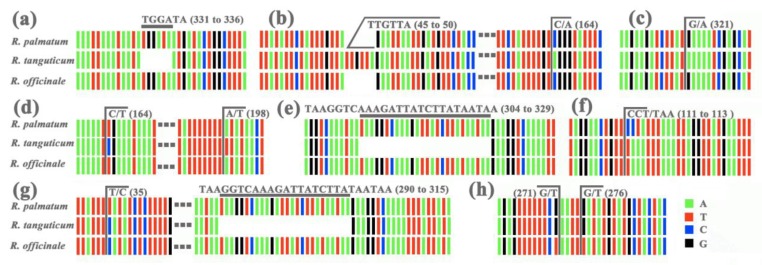
Base information of the identification sites of sequences obtained by chosen primer pairs for the three study species. (**a**) Primer pair 1; (**b**) Primer pair 7; (**c**) Primer pair 9; (**d**) Primer pair 10; (**e**) Primer pair 15; (**f**) Primer pair 17; (**g**) Primer pair 21; (**h**) Primer pair 6. For more detailed information on primer pairs see Table S3.

In order to distinguish these three species, reference to the chloroplast genome interspecific analysis, specific primer pairs were designed for the different regions, and the target fragments were amplified in the nearly one hundred samples (experiment with 30 samples of each species). Primer pair 1, used to amplify the *trnD-trnT* intergenic spacer region, can be employed to identify *R. tangutum* from the other two species because of its six bases deletion at 331 to 336 in the sequence alignment (Figure 5a). The intergenic spacer of *PsaA-ycf3* amplified by Primer pair 7 can distinguish *R. tangutum* for the base insertion (from site 45 to 50) and the base substitution (C to A at 164) (Figure 5b). Primer pair 9 and primer pair 10 were used to amplify the genes *rpoA* and *rpl16*, respectively. SNPs were found at site 321 (G to A) in *rpoA*, as well as 164 (T to C) and 198 (A to T) in *rpl16*, which can also be used to distinguish *R. tangutum* (Figure 5c,d). Primer pair 15, amplified in the *trnN-ycf1* intergenic spacer sequence, was also appropriate for the identification of *R. tangutum* due to the base deletion at 304 to 329 (Figure 5e). The region amplified by primer pair 17 has base substitutions from 111 to 113: CCT/TAA, so that it can be used to identify *R. palmatum* (Figure 5f). The amplified *trnN-ycf1* intergenic spacer from primer pair 21 was very specific, with a C to T substitution at 35, which can be used to identify *R. palmatum*, and a 26 base deletion at the site 290 to 315, which can be used to identify *R. tangutum* (Figure 5g). The *PsbD-trnT* intergenic spacer amplified by primer pair 6 was used to identify *R. officinale* with base substitutions at 271 (G to T) and 276 (G to T) (Figure 5h). The three species can be distinguished based on the substitution and deletion of bases in the target fragment amplified by primer pair 21.

## 3. Discussion

At present, DNA barcoding technology relies on chloroplast loci [18], such as *matK*, *rbcL*, *trnH-psbA*, *rpoB*, *rpoC1*, *atpF-atpH*, *psbK-psbI*, *ycf5*, and *trnL*, and has been discussed in detail [19,20]. These traditional single chloroplast loci typically lack sufficient variation; phylogenetic analyses of these chloroplast regions at higher taxonomic levels are meaningful, but chloroplast loci are not generally suitable for lower taxonomic levels. Because of the inherent deficiencies of single-locus DNA barcoding, a new method needs to be found to identify closely related species [21]. It has recently been suggested that the complete chloroplast genome contains as many sites of variation as mitochondrial regions in animals and may be used as a plant DNA barcode [22]. The chloroplast genome is now considered a species-level DNA barcode because it can greatly improve plant phylogenetic and population genetic analyses, facilitating the recovery of lineages as monophyletic at lower taxonomic levels [23]. Using the chloroplast genome as a plant DNA barcode can prevent identification failures caused by gene deletion and low PCR amplification success rate, and it can also solve the problem of sequence retrieval encountered in traditional barcode research [24,25]. The sequencing costs and obtaining high-quality DNA were once the main challenges of the enrichment of the chloroplast ultra-barcode database [26], but these challenges have been overcome by next-generation sequencing (NGS) combined with other technologies [27]. Thus, neither extraction methods nor sequencing capacity can be considered limiting factors for obtaining chloroplast genome data [28]. Currently, whole chloroplast genomes have been used as super molecular markers for species identification and phylogenetic analysis of closely related plant species [18,29,30]; medicinal plants, such as *Gynostemma pentaphyllum*, have also been analyzed by using chloroplast genomes [31]. In the present study, three closely related *Rheum* species that cannot be accurately identified by traditional barcodes (single-locus and multi-locus barcodes) were analyzed. The size, number of annotated genes, and the number of simple sequence repeats of their chloroplast genomes were different, and the results of the corresponding phylogenetic analysis showed the genomes can be effectively used to distinguish among these closely related species (Figure 3). The results further suggested the potential use of the chloroplast genome as a super barcode for the identification of closely related species.

The ultimate goal of DNA barcoding is to distinguish species rather than find a universal marker (Li et al., 2015). It is very expensive to sequence the chloroplast genome for each species, but a single gene locus was not suitable at the species level due to its modest discriminatory power, so we sought mutational hotspots to design primer pairs that can be used to identify the three different botanical origins of rhubarb. The *trnD-trnT* intergenic spacer, *psbD-trnT* intergenic spacer, *psaA-ycf3* intergenic spacer, *rpl16-rps3* intergenic spacer, *trnN-ycf1* intergenic spacer, *ndhF-rpl32* intergenic spacer, *trnN (GUU)-ycf1* intergenic spacer, *rpoA*, and *rpl16* were found to have existing stable mutation sites that can be used for the identification of rhubarb botanical origins. Therefore, a DNA fragment having sufficiently high mutation rate and being easily amplified is sought to be able to recognize a species in a given taxonomic group. The chloroplast genome is an effective approach to differentiate closely related plants, including most of the multi-original herbal medicines.

In the Chinese Pharmacopoeia 2015 edition, about one-quarter of the Chinese traditional medicines have multiple origins, meaning they could be derived from different species. Thus, the accurate identification of the different botanical origins of these multi-origin Chinese traditional medicines has become a focus of attention in society. Correct identifications ensure the safety of clinical medications and the control of drug quality. The present study laid the foundation of super barcode utilization in rhubarb, providing a molecular basis for precision medication, and lays the groundwork for the next investigation on these important medicinal species. This research has also provided a reference on the identification of the botanical origin of multi-origin medicinals.

## 4. Materials and Methods

### 4.1. Plant Materials and DNA Extraction

Fresh leaves of the three different botanical species (*R. officinale*, *R. palmatum*, *R. tangutum*) for chloroplast genome sequencing were collected from Qinghai, Sichuan, and Gansu Province, China (Table 1). The silica gel dried samples used for specific markers screening were collected from Gansu, Guizhou, Hebei, Henan, Hubei, Jilin, Qinghai and Sichuan Province, and thirty samples were collected for each of the species. The voucher specimens were deposited at the Hubei Institute for Drug Control and identified by Professor Ling Xiao. Total genomic DNA was extracted from leaves with a modified cetyltrimethylammonium bromide (CTAB) method [32,33]. The concentration of DNA was estimated by measuring A260 and A280 using an ND-2000 spectrometer (Nanodrop Technologies, Wilmington, DE, USA), samples were also visually examined by 1% agarose 1× TAE gel electrophoresis.

### 4.2. Sequencing, Assembly, and Annotation

The DNA integrity and quantity were analyzed by 1% agarose gel electrophoresis, a NanoDrop 2000C Spectrophotometer (Thermo Scientific, Waltham, MA, USA), Qubit^®^2.0 Fluorometer (Invitrogen, Carlsbad, CA, USA), and Agilent 2100 Bioanalyzer (Agilent, Santa Clara, CA, USA). Then the DNA was randomly fragmented into ~300 bp long fragments using an ultrasonicator (Bioruptor Pico, Denville, NJ, USA). After the sequencing libraries were constructed according to the manufacturer’s protocols (NEBNext^®^Ultra^TM^DNA Library Prep Kit for Illumina^®^, Beijing, China), sequencing was carried out on an Illumina HiSeq2000 high-throughput sequencer. The raw reads obtained were filtered using the NGS QC Toolkit [34], to omit the reads with more than 30% low quality bases (Q < 30) and those with more than 5% the amount of non-ATCG (N). The low quality regions of the reads were trimmed using trimmingReads.pl. Clean data were stored for next analysis. All the clean reads were collected as a pool for chloroplast genome assembly, and Geneious 9.1.4 (Biomatters Ltd., Auckland, New Zealand) [35] with BLAST 2.0.3+ (National Institutes of Health, Bethesda, MD, USA) [36] was employed to assemble the genomes. Four junctions between the inverted repeat (IR) and large single-copy/small single-copy (LSC/SSC) regions were confirmed by PCR amplification and Sanger sequencing.

DOGMA (available online: http://dogma.ccbb.utexas.edu/) [37] and CPGAVAS [38] were used for annotating the chloroplast genome to compare them between the three study species and further confirmation was performed using BLAST and DOGMA [37]. The tRNA genes were identified by tRNAscanSE [39]. Circular genome maps of the three different botanical origins of rhubarb were illustrated with the Organellar Genome DRAW tool [40]. The characteristics of chloroplast genomic sequences were determined using MEGA7 [41].

### 4.3. Identification of Repeated Sequences

Simple sequence repeats (SSR) and scattered repeats in all three species were also investigated. Simple sequence repeats were searched by MISA [42] with thresholds of 8, 4, 3, 3, 3, and 3 for mono-, di-, tri-, tetra-, penta-, and hexa-nucleotide, respectively. Scattered repeats were detected using the program REPuter [43] with parameters set as the similarity percentage of scattered repeat copies was at least 90%, hamming distance = 3, and the parameter of minimal repeat size was 30 bp.

### 4.4. Phylogenetic Reconstruction

In order to explore the phylogenic relationships of the three species, and to assess the identification efficiency of the chloroplast genomes, chloroplast genomes of *Lilium tsingtauense* (KU230438), *Anemarrhena asphodeloides* (KX931449), *Najas flexiles* (NC_021936), *Elaeis guineensis* (NC_017602), *Zingiber spectabile* (JX088661), *Bambusa oldhamii* (FJ970915), *Carludovica palmate* (NC_026786), *Amborella trichopoda* (NC_005086), *Salvia miltiorrhiza* (JX312195), *Acorus calamus* (AJ879453), *Panax ginseng* (KF431956), *Oryza alta* (KF359913), *Fagopyrum esculentum* (NC_010776.1), *Dendrobium officinale* (KJ862886), *Yucca schidigera* (NC_032714), *Petrosavia stellaris* (KF482381), *Xiphidium caeruleum* (JX088669), *Aconitum barbatum* (KT964698), *Rheum wittrockii* (NC_035950.1), and *Fagopyrum tataricum* (NC_027161.1), a total of 20 genome sequences, were downloaded from NCBI and aligned using ClustalW2 [44] and MAFFT [45]. Phylogenetic relationships were analyzed using the neighbor-joining method in MEGA 7.0.26 by a Poisson model for nucleotide sequence, with Poisson correction, pairwise deletion of gaps, and bootstrap analysis with 1000 replications. *Amborella trichopoda* was set as outgroup.

### 4.5. Candidate Identification Sequence Screening

Based on the whole genome sequences of the three chloroplasts, 21 pairs of primers toward the variable regions were designed for PCR amplification (Table S3). PCR amplification reactions were performed in a final volume of 25 μL. Each reaction mixture contained 10× PCR buffer 2.5 μL, 25 mM MgCl_2_ 2 μL, 2.5 mM dNTP 2 μL, Taq (5 U/µL) 0.2 μL, forward primers (10 μM) 1 μL, reverse primers (10 μM) 1 μL, template DNA 2 μL, add water to 25 μL. The PCR protocol followed the profile of 95 °C for 4 min, 35 cycles of 94 °C for 30 s, 49 °C~55 °C for 1 min, and at 72 °C for 1 min; 72 °C for 10 min, and subsequent storage at 4 °C. Ninety silica gel dried samples from different localities were collected as the sequence screening samples. After the amplified products were detected by 1% agarose gel electrophoresis, the PCR products showing a single crisp band were purified by DNA product purification kit (Cat: D1300, Solarbio, Beijing, China), then they were bidirectionally sequenced by the ABI3730XL sequencer (Applied Biosystems Co., Shanghai, China). The peak maps obtained by sequencing were aligned using the CodonCode Aligner V3.7.1 (CodonCode Co., Centerville, MA, USA), and the primer regions and low-mass regions were removed to obtain candidate sequences for identification. Phylogenetic analyses were conducted to distinguish whether the candidate regions can be used to identify the three study species employing MEGA7 program (National Institutes of Health, Bethesda, MD, USA).

## Figures and Tables

**Table 1:** The basic characteristics of chloroplast genomes of the three *Rheum* species.

	*R. officinale*	*R. palmatum*	*R. tanguticum*
Location	Qinghai	Sichuan	Gansu
Accession number in GenBank	MH572012	KR816224	MH572013
Total clean read	820,613 kb	644,941 kb	685,879 kb
N50 of contigs (bp)	86,523	86,483	86,439
Total chloroplast DNA size (bp)	161,093	161,541	161,053
LSC size (bp)	86,609	86,518	86,604
IR size (bp)	30,956	30,956	30,961
SSC size (bp)	12,750	13,111	13,147
Total number of genes	131	130	129
Number of different protein-coding genes	92	92	92
Number of different tRNA genes	31	30	29
Number of different rRNA genes	8	8	8
GC content (%)	37.3	37.3	37.3
GC content of LSC (%)	35.3	35.4	35.4
GC content of IR (%)	41.1	41	41.1
GC content of SSC (%)	32.5	32.5	32.6

LSC: large single-copy region; IR: inverted repeats; SSC: small single-copy region.

**Table 2:** A list of genes found in the chloroplast genomes of the three Rheum species including copy number and introns included.

Group of Genes	Name of Gene
Transfer RNAs (31)	*trnC-GCA*, *trnD-GTC*, *trnE-TTC*, *trnF-GAA*, *trnfM-CAT*, *trnG-GCC*, *trnH-GTG*, *trnI-CAT* (x2), *trnI-GAT* *, *trnL-CAA* (x2), *trnL-TAG*, *trnL-TAG* *, *trnM-CAT*, *trnN-GTT* (x2), *trnP-TGG*, *trnQ-TTG*, *trnR-ACG* (x2), *trnR-TCT*, *trnS-GGA*, *trnS-GCT*, *trnS-TGA*, *trnT-GGT*, *trnT-TGT*, *trnV-GAC* (x2), *trnW-CCA*, *trnY-GTA*,
photosystem I (5)	*psaA*, *psaB*, *psaC*, *psaI*, *psaJ*
Assembly/stability of photosystem I (2)	ycf3 **, *ycf4*
photosystem II (15)	*psbA*, *psbB*, *psbC*, *psbD*, *psbE*, *psbF*, *psbH*, *psbI*, *psbJ*, *psbK*, *psbL*, *psbM*, *psbN*, *psbT*, *psbZ*
Maturase (1)	*matK*
Ribosomal protein (25)	*rps16*, *rps2*, *rps14*, *rps4*, *rpl33*, *rps18*, *rpl20*, *rps11*, *rpl36*, *rps8*, *rpl14*, *rpl16*, *rps3*, *rpl22*, *rps19*, *rps15*, *rps12* (x3), *rpl2* * (x2), *rpl23* (x2), *rps7* (x2),
cytochrome b6/f complex (6)	*petA*, *petB*, *petD*, *petG*, *petL*, *petN*
ATP synthase (6)	*atpA*, *atpB*, *atpE*, atpF *, *atpH*, *atpI*
RNA polymerase (4)	*rpoC2*, rpoC1 *, *rpoB*, *rpoA*
NADH dehydrogenase (13)	ndhA *, ndhB * (x2), *ndhC*, *ndhD*, *ndhE*, *ndhF*, *ndhG*, *ndhH*, *ndhI*, *ndhJ*, *ndhK* (x2)
Rubisco large subunit (1)	*rbcL*
Acetyl-CoA carboxylase (1)	*accD*
envelope membrane protein (1)	*cemA*
ATP-dependent protease subunit (1)	clpP **
translation initiation factor (1)	*infA*
Conserved reading frames (ycfs) (8)	*ycf2* (x2), *ycf15* (1N) (x2), *ycf15* (x2), *ycf1* (1N) (x2)
Ribosomal RNAs (8)	*rrn16S* (x2), *rrn23S* (x2), *rrn4.5S* (x2), *rrn5S* (x2)
c-type cytochrome biogenesis (1)	*ccsA*

* contains one intron; ** contains two introns; Numbers in brackets behind name of gene group give number of repetitive genes; *trnI-GAT* * exists in *R. officinale*.

**Table 3:** *R. officinale* chloroplast genome SSR distribution.

SSR nr.	SSR Type	SSR	Size	Star	End	Location
2	p1	(A)10	10	1883	1892	CNS
3	p1	(T)11	11	2053	2063	CNS
4	p4	(TGAT)3	12	2688	2699	*matK*
5	p1	(T)12	12	3040	3051	*matK*
6	p1	(T)11	11	3505	3515	*matK*
8	p1	(A)12	12	4763	4774	CNS
10	p1	(T)10	10	5538	5547	CNS
16	p1	(A)12	12	8117	8128	CNS
23	p4	(GTCT)3	12	12207	12218	*atpA*
32	p1	(T)11	11	19306	19316	*rpoC2*
35	p2	(AT)5	10	20683	20692	*rpoC2*
49	p1	(T)10	10	33669	33678	CNS
50	p1	(A)12	12	34263	34274	CNS
57	p1	(A)10	10	39162	39171	CNS
62	p1	(T)10	10	45369	45378	CNS
63	p3	(AAT)4	12	46315	46326	CNS
65	p4	(TTGG)3	12	46894	46905	CNS
87	p4	(TATT)3	12	61268	61279	CNS
90	p2	(TA)5	10	63994	64003	*petA*
97	p1	(A)10	10	67687	67696	*petL*-CNS
116	p1	(T)15	15	82003	82017	CNS
120	p1	(T)12	12	86283	86294	*rpl22*
125	p1	(A)10	10	89370	89379	*ycf2*
129	p3	(CTT)4	12	92498	92509	*ycf2*
151	p1	(A)16	16	114057	114072	*ycf1*
158	p1	(A)10	10	117661	117670	*ndhF*
165	p1	(T)10	10	120389	120398	CNS
171	p4	(AATA)3	12	122749	122760	*ndhD*
174	p2	(AT)5	10	124108	124117	CNS
175	p1	(A)10	10	125400	125409	*ndhG*
183	p1	(T)16	16	133545	133560	*ycf1*
205	p3	(AAG)4	12	155108	155119	*ycf2*
209	p1	(T)10	10	158238	158247	*ycf2*

**SSR**: Simple sequence repeats; CNS: non-coding sequences.

## Supplementary Materials

The following are available online, Supplementary Table S1: *R. palmatum* chloroplast genome SSR distribution. Supplementary Table S2: *R. tanguticum* chloroplast genome SSR distribution. Supplementary Table S3: Primer pairs information used to distinguish among species identification candidate regions.
