# Supplementary material for: Comparative Chloroplast Genome Analysis of Rhubarb Botanical Origins and the Development of Specific Identification Markers
Source: Molecules. 2018 Oct 30;23(11):2811. doi: 10.3390/molecules23112811 (PMC6278470; doi:10.3390/molecules23112811)
Supplement: Supplementary file 1 [file molecules-23-02811-s001.pdf]

Table S1 *R. palmatum* chloroplast genome SSR distribution

SSR nr.	SSR type	SSR	size	start	end	Location
2	p1	(A)10	10	1890	1899	CNS
3	p4	(TGAT)3	12	2695	2706	*matK*
4	p1	(T)12	12	3047	3058	*matK*
5	p1	(T)10	10	3513	3522	*matK*
15	p1	(A)11	11	8096	8106	CNS
19	p1	(A)10	10	9546	9555	CNS
23	p4	(GTCT)3	12	12166	12177	*atpA*
32	p1	(T)11	11	19265	19275	rpoC2
35	p2	(AT)5	10	20642	20651	rpoC2
49	p1	(T)10	10	33658	33667	CNS
50	p1	(A)12	12	34252	34263	CNS
62	p1	(T)11	11	45355	45365	*ycf3*
63	p3	(AAT)4	12	46302	46313	*ycf3*
65	p4	(TTGG)3	12	46881	46892	CNS
87	p4	(TATT)3	12	61257	61268	CNS
90	p2	(TA)5	10	63984	63993	*petA*
97	p1	(A)10	10	67677	67686	*petL*-CNS
115	p1	(T)10	10	80646	80655	*rpoA*
117	p1	(T)15	15	82015	82029	CNS
126	p1	(A)10	10	89365	89374	*ycf2*
130	p3	(CTT)4	12	92493	92504	*ycf2*
152	p1	(A)16	16	114079	114094	*ycf1*
159	p1	(A)10	10	117683	117692	*ndhF*
172	p4	(AATA)3	12	123133	123144	*ndhD*
176	p2	(AT)5	10	124492	124501	CNS
177	p1	(A)10	10	125821	125830	*ndhG*
178	p4	(ATTT)3	12	126224	126235	CNS
185	p1	(T)16	16	133966	133981	*ycf1*
207	p3	(AAG)4	12	155556	155567	*ycf2*
211	p1	(T)10	10	158686	158695	*ycf2*

SSR: Simple sequence repeats; CNS: non-coding sequences.

Table S2 *R. tanguticum* chloroplast genome SSR distribution

SSR nr.	SSR type	SSR	size	start	end	Location
3	p4	(TGAT)3	12	2687	2698	*matK*
4	p1	(T)12	12	3039	3050	*matK*
5	p1	(T)10	10	3505	3514	*matK*
9	p1	(T)10	10	5533	5542	CNS
15	p1	(A)12	12	8079	8090	CNS
19	p1	(A)10	10	9527	9536	CNS
23	p4	(GTCT)3	12	12180	12191	*atpA*
26	p1	(T)11	11	14489	14499	CNS
32	p1	(T)11	11	19277	19287	*rpoC2*
35	p2	(AT)5	10	20654	20663	*rpoC2*
50	p2	(TA)5	10	33631	33640	CNS
63	p1	(T)10	10	45330	45339	ycf3
64	p3	(AAT)4	12	46276	46287	*ycf3*
66	p4	(TTGG)3	12	46855	46866	CNS
88	p4	(TATT)3	12	61194	61205	CNS
90	p2	(TA)5	10	63920	63929	*petA*
94	p1	(A)13	13	66843	66855	CNS
97	p1	(A)10	10	67600	67609	*petL*-CNS
100	p1	(T)10	10	68576	68585	CNS
116	p1	(T)15	15	81935	81949	CNS
125	p1	(A)10	10	89286	89295	*ycf2*
129	p3	(CTT)4	12	92414	92425	*ycf2*
150	p1	(A)16	16	113978	113993	*ycf1*
157	p1	(A)10	10	117582	117591	*ndhF*
158	p3	(TTA)4	12	117748	117759	*ndhF*
169	p4	(AATA)3	12	122704	122715	*ndhD*
173	p2	(AT)5	10	124063	124072	CNS
174	p1	(A)10	10	125355	125364	*ndhG*
175	p4	(ATTT)3	12	125758	125769	CNS
182	p1	(T)16	16	133500	133515	*ycf1*
203	p3	(AAG)4	12	155068	155079	*ycf2*
207	p1	(T)10	10	158198	158207	*ycf2*

SSR: Simple sequence repeats; CNS: non-coding sequences.

Table S3 Primer pairs information used to distinguish among species identification candidate regions

Primer name	Primer sequence	Location	Identification
F_PRIMER1:	5'-TGATGCGATCCAAGGTATCC-3'	*trnD-trnT* intergenic spacer	*R. tanguticum*
R_PRIMER1:	5'-TGATCCCCTTTATCATCCCGA-3'		
F_PRIMER2:	5'-TGCATTCTTTCCTCGACCCA-3'	*psbD-trnT* intergenic spacer	—
R_PRIMER2:	5'-TTTCGAACACCTCATAGCGC-3'		
F_PRIMER3:	5'-AAAGTAAGTGGACCTGGCCT-3'	*psbD-trnT* intergenic spacer	—
R_PRIMER3:	5'-TGGGTCGAGGAAAGAATGCA-3'		
F_PRIMER4:	5'-TGGGGTGGAAAATACATGCG-3'	*psbD-trnT* intergenic spacer	—
R_PRIMER4:	5'-ACCTCATAGCGCGGAGTC-3'		
F_PRIMER5:	5'-AAAGTAAGTGGACCTGGCCT-3'	*psbD-trnT* intergenic spacer	—
R_PRIMER5:	5'-TCGCATGTATTTTCCACCCC-3'		
F_PRIMER6	5'-TGCATTCTTTCCTCGACCCA-3'	*psbD-trnT* intergenic spacer	*R. officinale*

R_PRIMER6	5’-GTCCCTCCGTAACCAGTCAT-3’		
F_PRIMER7	5’-CCAAATCATGCGAGCAGTCA-3’	*psaA-ycf3* intergenic spacer	*R. tanguticum*
R_PRIMER7	5’-TCGATCAAGCCGCTGAGTAT-3’		
F_PRIMER8	5’-AGAGGTGAGGTATTAATTGCTCA-3’	*psaA-ycf3* intergenic spacer	—
R_PRIMER8	5’-CTCTCAAGTACGGTTCTAAGGGA-3’		
F_PRIMER9	5’-TGCTTTTCGAGAGTGTCCAA-3’	*rpoA* gene	*R. tanguticum*
R_PRIMER9	5’-TGCTGTATTCATGCCTGTTCG-3’		
F_PRIMER10	5’-TGCCGAAACAAATACGATTACCT-3’	*rpl16* gene	*R. tanguticum*
R_PRIMER10	5’-GAAAGAGTAAATATTCGCCCGC-3’		
F_PRIMER11	5’-TGACTGCTTCCTTGGATCCA-3’	*rpl16-rps3* intergenic spacer	*R. palmatum*
R_PRIMER11	5’-ACTGAACAGGCTGATACAAAAGG-3’		
F_PRIMER12	5’-CGTGCAATTTCTTTTCCGTCG-3’	*rps3* gene	—
R_PRIMER12	5’-TCTAGTGGGGCAATATGGGAC-3’		

F_PRIMER13	5’-ATTCACACTCCACGGTCTGA-3’	*ycf2*	—
R_PRIMER13	5’-ATTTGGCGAGCTCAGATGTG-3’		
F_PRIMER14	5’-CTCAAAGGGGCGTGTAAACA-3’	*trnV-GAC*	—
R_PRIMER14	5’-CCCACGAGCCTCTTATCCAT-3’		
F_PRIMER15	5’-AAACTTCTTCGTAGCGGCTC-3’	*trnN-ycf1* intergenic spacer	*R. tanguticum*
R_PRIMER15	5’-GAAGGACGAGATAGGAGGGC-3’		
F_PRIMER16	5’-ACTGAATGGGTAAGTCGCCA-3’	*trnL(CAA)*	—
R_PRIMER16	5’-AGCAATACTCCAACACCCCA-3’		
F_PRIMER17	5’-CCCGCTCCGATTAACAAAGG-3’	*ndhF-rpl32* intergenic spacer	*R. palmatum*
R_PRIMER17	5’-CAAAAGTAAAGTCTTGGCTAGCA-3’		
F_PRIMER18	5’-TCGTTAAGTAGGTAAGAGCAGCT-3’	*ndhF-rpl32* intergenic spacer	—
R_PRIMER18	5’-TGCTGCCGAATGTCCTTTTC-3’		
F_PRIMER19	5’-AGACACGCTGCTCTTAGGAA-3’	*trnL(UAG)*	—

R_PRIMER19	5’-TGCCCCGAATAAATCCAACG-3’		
F_PRIMER20	5’-AGGTATTCTTTCAGGAGCCGT-3’	*ccsA-ndhD* intergenic spacer	—
R_PRIMER20	5’-TTTGATTCAGGTCCGCGAGA-3’		
F_PRIMER21	5’-GAAGGACGAGATAGGAGGGC-3’	*trnN(GUU)-ycf1* intergenic spacer	Distinguish three species
R_PRIMER21	5’-CCCTTCCATGCCTCATTTCA-3’		
